# Childhood Lead Exposure in Primary Health Care Settings in Indonesia: A Cross-Sectional Study in Bogor City and Bogor Regency

**DOI:** 10.3390/ijerph23081046

**Published:** 2026-08-12

**Authors:** Anna Suraya, Maryuni, Desi Aryanie, Lelitasari, Uci Sulandari, Maya Setyawati, Sari Narulita, Renan Prasta Jenie, Sri Utami Ningsih, Eni Kusrini, Agung Cahyono Triwibowo, Then Suyanti, Rooswanti Soeharno, Mrunal Shetye

**Affiliations:** 1 Faculty of Health Science and Technology, Binawan University, Jakarta 13630, Indonesia; maryuni@binawan.ac.id (M.); desi.aryani@binawan.ac.id (D.A.); lelitasari@binawan.ac.id (L.); uci.sulandri@binawan.ac.id (U.S.); maya.setyawati@yahoo.com (M.S.); sari@binawan.ac.id (S.N.); sriutami.parahita@gmail.com (S.U.N.); agungcahyonot@binawan.ac.id (A.C.T.); 2 Faculty of Business and Management Science, Binawan University, Jakarta 13630, Indonesia; renan.prasta@binawan.ac.id; 3 Ministry of Health of Republic Indonesia, Jakarta 12950, Indonesia; thensuyanti@yahoo.com; 4 Unicef Indonesia, Jakarta 12920, Indonesia; rsoeharno@unicef.org (R.S.); mshetye@unicef.org (M.S.)

**Keywords:** lead exposure, blood lead level, geographic mapping, public health screening

## Abstract

**Highlights:**

**Public health relevance—How does this work relate to a public health issue?**

- Childhood lead exposure remains an important environmental health concern in Indonesia, with 21.9% of children aged 1–5 years in this study having elevated blood lead levels (BLL) ≥ 5 µg/dL.
- The study identifies clear geographic clustering of elevated blood lead levels, particularly in Bogor Regency and among children living near lead-processing activities, indicating localized environmental exposure risks.

**Public health significance—Why is this work of significance to public health?**

- This study provides evidence from routine primary health care settings, addressing an important gap in Indonesia where most previous studies focused on selected high-risk communities or environmental hotspots.
- The findings show that primary health care–based screening can help detect vulnerable children and generate actionable data for targeted environmental and public health responses.

**Public health implications—What are the key implications or messages for practitioners, policy makers and/or researchers in public health?**

- Lead exposure management should be integrated into routine child health services, supported by strengthened laboratory capacity, referral pathways, caregiver counselling, and follow-up mechanisms.
- Future programs and studies should combine health facility screening with environmental investigation to confirm exposure sources and guide cross-sectoral interventions involving health and environmental authorities.

**Abstract:**

(1) Background: Lead exposure is an important preventable environmental health concern among children, especially in low- and middle-income countries. In Indonesia, evidence from routine health service settings remains limited. (2) Methods: This cross-sectional study assessed blood lead levels (BLLs) and associated factors among children aged 1–5 years attending 12 primary health care facilities in Bogor City and Bogor Regency between December 2025 and January 2026. A total of 420 children were recruited consecutively during routine services. Environmental and household risk factors were collected using structured questionnaires, and venous blood samples were analyzed using inductively coupled plasma mass spectrometry. Elevated BLL was defined as ≥5 µg/dL. (3) Results: The mean BLL was 2.24 µg/dL, and 21.9% of children had elevated BLLs. Prevalence was higher in Bogor Regency than in Bogor City (41.2% vs. 2.4%; *p* < 0.001) and among children residing near lead-processing activities (44.2% vs. 16.2%; *p* < 0.001). One child had a BLL of 66.82 µg/dL. Results were returned to caregivers and facilities; children with BLL ≥ 5 µg/dL received counselling, exposure-reduction advice, nutritional support, and follow-up planning, while the child with BLL > 45 µg/dL was urgently referred for confirmatory assessment and specialist management. (4) Conclusions: Primary healthcare–based screening can identify vulnerable children and trigger clinical, nutritional, environmental, and referral responses, although longitudinal follow-up and environmental source testing are needed.

## 1. Introduction

Lead exposure remains a major global environmental health concern, particularly among children, for whom no safe Blood Lead Level (BLL) has been identified. Even low-level exposure is associated with adverse neurodevelopmental, behavioral, and physical health outcomes [1,2,3,4]. Globally, lead exposure continues to disproportionately affect children in low- and middle-income countries (LMICs), where rapid urbanization, informal industries, and weak environmental regulation contribute to persistent exposure risks [1,5].

In Indonesia, the burden of lead exposure is substantial and increasingly recognized as a critical public health issue. Recent estimates suggest that more than 8 million children have BLL exceeding 5 µg/dL, the threshold requiring clinical and environmental intervention [6,7]. Earlier studies have consistently documented elevated BLL in Indonesian children since the 1990s, with particularly high levels observed in areas near informal used lead–acid battery (ULAB) recycling, mining, and other environmentally hazardous activities [8,9,10,11,12]. For example, children living near ULAB recycling sites in Bogor Regency have shown markedly elevated BLL, while studies in mining areas have demonstrated associations between lead exposure and impaired growth outcomes [8,13].

Despite growing evidence, important gaps remain in understanding the distribution, determinants, and health correlates of lead exposure within routine health system settings. Most prior studies in Indonesia have focused on specific high-risk populations or environmental hotspots, with limited integration of clinical, environmental, and health outcome data.

Within this context, the present study—conducted within a broader implementation research program—examines BLL among children aged 1–5 years attending primary health care facilities in Bogor City and Bogor Regency. By combining facility-based quantitative data with contextual environmental observations across two contrasting settings, the study describes the distribution, correlates and geographic concentration of lead exposure in routine service settings. The findings are intended to inform health system–integrated strategies for childhood lead exposure detection and response in Indonesia and similar LMIC settings.

## 2. Materials and Methods

This study was initiated, designed and funded by UNICEF, with strategic support from the Ministry of Health of Indonesia. Implementation was conducted in collaboration with local health authorities and the primary health care facility network, ensuring integration within routine service delivery. Binawan University served as the lead academic institution, coordinating the overall implementation, data management and analysis.

### 2.1. Study Design and Setting

This study was conducted as part of Phase 1 of a phased implementation research program designed to evaluate the integration of the National Guideline on Clinical Management of Lead Exposure in Children and Pregnant Women into routine health services in Indonesia. Within this framework, the present study specifically aimed to describe BLL distribution in relation to environmental exposure sources across different geographic contexts, namely Bogor City and Bogor Regency. These two areas were selected due to their contrasting exposure profiles, with Bogor Regency characterized by the presence of informal lead-related activities, and Bogor City representing a relatively lower-exposure urban setting.

A cross-sectional study design was employed across 12 primary health centers (PHCs)—six in Bogor City and six in Bogor Regency—selected to represent diverse environmental exposure contexts and support comparative analysis between settings. In addition, environmental field observations were conducted to provide contextual information supporting the interpretation of facility-level and geographic patterns of BLL, although these observations were not analyzed as formal qualitative data. Bogor Regency was selected because previous studies have documented elevated BLL among children living near informal used lead–acid battery (ULAB) recycling activities. Bogor City was selected as a contrasting urban setting with lower documented environmental lead exposure. We have also added information regarding socioeconomic characteristics of the study population and known or suspected environmental lead sources in each area.

### 2.2. Participants and Recruitment

The study population included children aged 1–5 years attending participating primary health care facilities for routine services, including immunization, growth monitoring, and outpatient care, between December 2025 and January 2026. Participating facilities were located within the designated study areas and provided maternal and child health services, with the capacity and commitment to implement study standard operating procedures, participate in training and supervision activities, and conduct specimen storage and transport. Facilities undergoing major renovation, lacking minimum infrastructure for screening and specimen collection, or involved in other intensive implementation research projects were excluded.

Each facility recruited approximately 35 children, resulting in a total sample of 420 participants. The target sample size per facility was determined based on recruitment feasibility during the pilot implementation period, facility service volume, staff availability, and laboratory and logistical capacity.

Participants were recruited consecutively during routine service delivery. Inclusion criteria were children aged 1–5 years whose caregivers provided informed consent for participation, including blood sample collection. Children whose caregivers declined participation or blood collection were excluded from the study.

### 2.3. Capacity Building

Prior to implementation, health care workers received training based on the Ministry of Health (MoH) Training Curriculum and module on lead exposure management for children and pregnant women. The training covered risk screening, safe blood collection, clinical management pathways, and reporting procedures.

### 2.4. Data Collection

Data collection was conducted by health facility staff who had participated in prior training on the implementation of the national guideline.

Exposure source information was obtained using structured questionnaires adapted from the National Guideline on Clinical Management of Lead Exposure in Children and Pregnant Women. Variables included caregiver-reported proximity to lead-processing activities, informal use of ULAB recycling, mining or industrial activities, household lead-related occupations, peeling paint, housing conditions, and selected consumer products. “Area” represented the administrative study setting, classified as either Bogor City or Bogor Regency, whereas “proximity” referred to the caregiver-reported presence of lead-processing activities within approximately 2.5 km of the child’s residence. These variables captured different contextual dimensions: area reflected broader geographic and administrative differences, while proximity reflected a more localized potential exposure source. Although conceptually distinct, the two variables were expected to be related and were therefore interpreted cautiously in the multivariable analysis. Building age, paint lead concentration, and lead concentrations in water, soil, dust, and household products were not measured.

Anthropometric measurements and hemoglobin levels (as an indicator of anemia) were assessed using standard procedures routinely applied in child health services. Wasting was defined using the applicable weight-for-height/length indicator. Neurodevelopmental, cognitive, behavioral, renal, neurologic, hearing, and other longer-term outcomes were outside the scope of routine data collection and were not assessed.

### 2.5. BLL Measurement

BLL testing was performed using venous blood samples collected by trained personnel following contamination-minimizing procedures. Samples were transported under controlled conditions and analyzed at the DKI Jakarta Regional Health Laboratory using inductively coupled plasma mass spectrometry (ICP-MS; Agilent 7800, Agilent Technologies, Santa Clara, CA, USA). The analysis followed the laboratory’s internal method for lead in whole blood. Briefly, 0.5 mL of whole blood was digested with concentrated HNO_3_ at 90 °C for 1 h, followed by addition of concentrated H_2_O_2_ and further heating for 2 h. After cooling, samples were diluted to 10 mL with Milli-Q water (MilliporeSigma, Burlington, MA, USA), homogenized, filtered, and analyzed by ICP-MS (ICP-MS; Agilent 7800, Agilent Technologies, Santa Clara, CA, USA).

Calibration was performed using Pb standard solutions prepared in 10% HNO_3_. The method detection limit was 0.23 µg/dL; values below this limit were reported as non-detectable. Quality control included Lypochek Whole Blood Metal Control (Bio-Rad, Hercules, CA, USA) and internal spike-recovery checks, with acceptable recovery defined as 80–110%.

### 2.6. Data Analysis

Data were analyzed using descriptive and inferential statistics. Continuous variables were summarized using means, standard deviations, medians, and ranges, while categorical variables were presented as frequencies and percentages. Missing data for anemia and wasting were handled using available-case analysis, and no imputation was performed.

BLL distribution was analyzed overall and by facility. The proportion of children with elevated BLL (≥5 µg/dL) was calculated. For bivariate analysis, Pearson’s chi-square test was used when expected cell counts were adequate, and Fisher’s exact test was used for sparse tables. Multivariable binary logistic regression was conducted to estimate adjusted odds ratios and 95% confidence intervals. Area and proximity to lead-processing activities were retained because they represented administrative context and household-level reported proximity, respectively; however, their conceptual overlap and likely correlation require cautious interpretation. Formal variance inflation factor diagnostics and facility-clustered sensitivity analyses were not performed in this Phase 1 analysis.

### 2.7. Communication of Results and Follow-Up Actions

BLL results were communicated to participating primary health centers and caregivers. In accordance with the national guideline, all children with BLL ≥ 5 µg/dL received caregiver counselling on potential household and environmental sources, hygiene and exposure-reduction measures, and nutrition emphasizing adequate iron and calcium. The implementation program also provided calcium-rich milk powder for two servings daily for three months and established follow-up plans through the primary health center. The child with BLL ≥ 45 µg/dL was categorized as requiring urgent action and was referred for prompt clinical assessment, evaluation for possible ingestion and other complications, specialist consultation, and household/environmental investigation. The ≥45 µg/dL threshold was selected from the WHO and Indonesian clinical guidelines, which recommend chelation therapy for children at or above this concentration after clinical assessment and removal from ongoing exposure [7]. Chelation was considered within the referral pathway; the study does not report chelation as completed because treatment delivery and longer-term outcomes were outside the cross-sectional analysis period.

### 2.8. Ethical Considerations

Ethical approval for this study was obtained from the Ethics Committee of Binawan University (No: 629/KEPK-UBN/XI/2025). Written informed consent was obtained from parents or caregivers prior to participation, including consent for blood sample collection. All data were anonymized prior to analysis to ensure confidentiality.

### 2.9. Artificial Intelligence (AI) Use Disclosure

The authors used AI-based tools (The GPT-5.6 Thinking model, OpenAI, San Francisco, CA, USA) to assist in drafting sections of the manuscript, improving English grammar, and enhancing clarity of expression. All content was critically reviewed, revised, and approved by the authors, who take full responsibility for the accuracy and integrity of the manuscript.

## 3. Results

### 3.1. Distribution, Determinants, and Health Correlates of Childhood BLL

#### 3.1.1. Participant Characteristics

A total of 420 children under five years of age were included in the main analysis. Slightly more than half of the children were male (52.6%), while 46.9% were female. Regarding socioeconomic status, the majority of participants’ parents reported a monthly income below IDR 3 million (59.0%). In terms of environmental risk factors, 20.5% of participants resided near lead-processing activities, including informal used lead–acid battery (ULAB) recycling. Most participants lived in generally adequate housing conditions (90.2%); however, 45% reported peeling paint within the household. With regard to consumer product use, most parents (97.4%) reported not using cosmetics that were unlabeled or not confirmed as safe. Aluminum cookware was commonly used in households, with 34.8% reporting its use. Additionally, 12.4% had household members engaged in lead-related occupations (Table 1).

#### 3.1.2. Blood Lead Level

**Figure 1:**
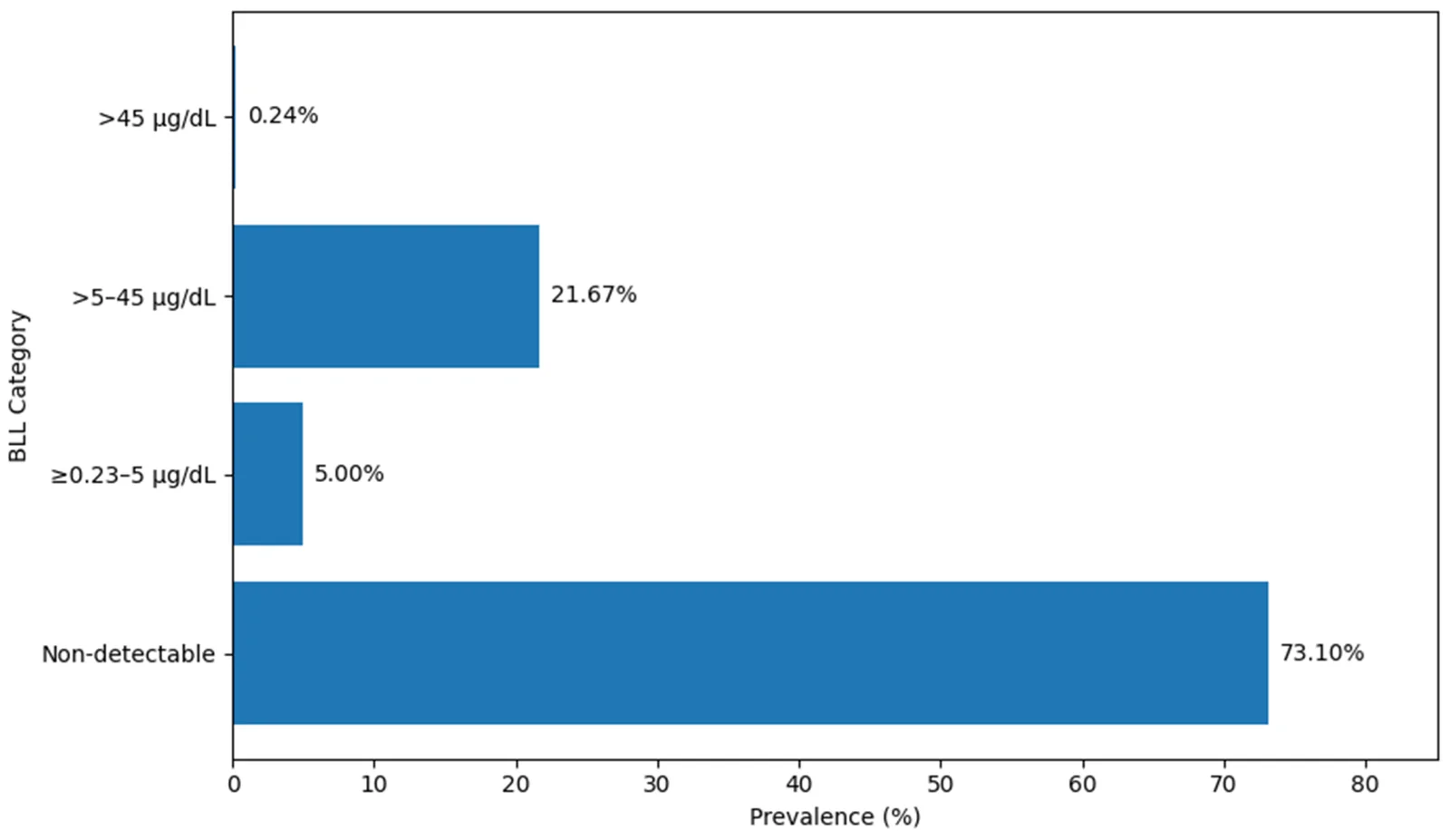
Prevalence of BLL categories among children aged 1–5 years.

The mean BLL was 2.24 µg/dL, with a standard deviation of 5.12 µg/dL, while the median and mode were 0.00 µg/dL, reflecting a strongly right-skewed distribution with many non-detectable values and a smaller group of children with higher exposure. The prevalence of elevated BLL (≥5 µg/dL) was 21.9% and a very small proportion (0.2%) exhibited BLL > 45 µg/dL. Notably, the child with the highest recorded BLL (66.82 µg/dL) was reported to have a history of recurrent anemia, highlighting a potential clinical manifestation associated with severe lead exposure (Figure 1).

#### 3.1.3. Geographic/Facility Distribution

**Figure 2:**
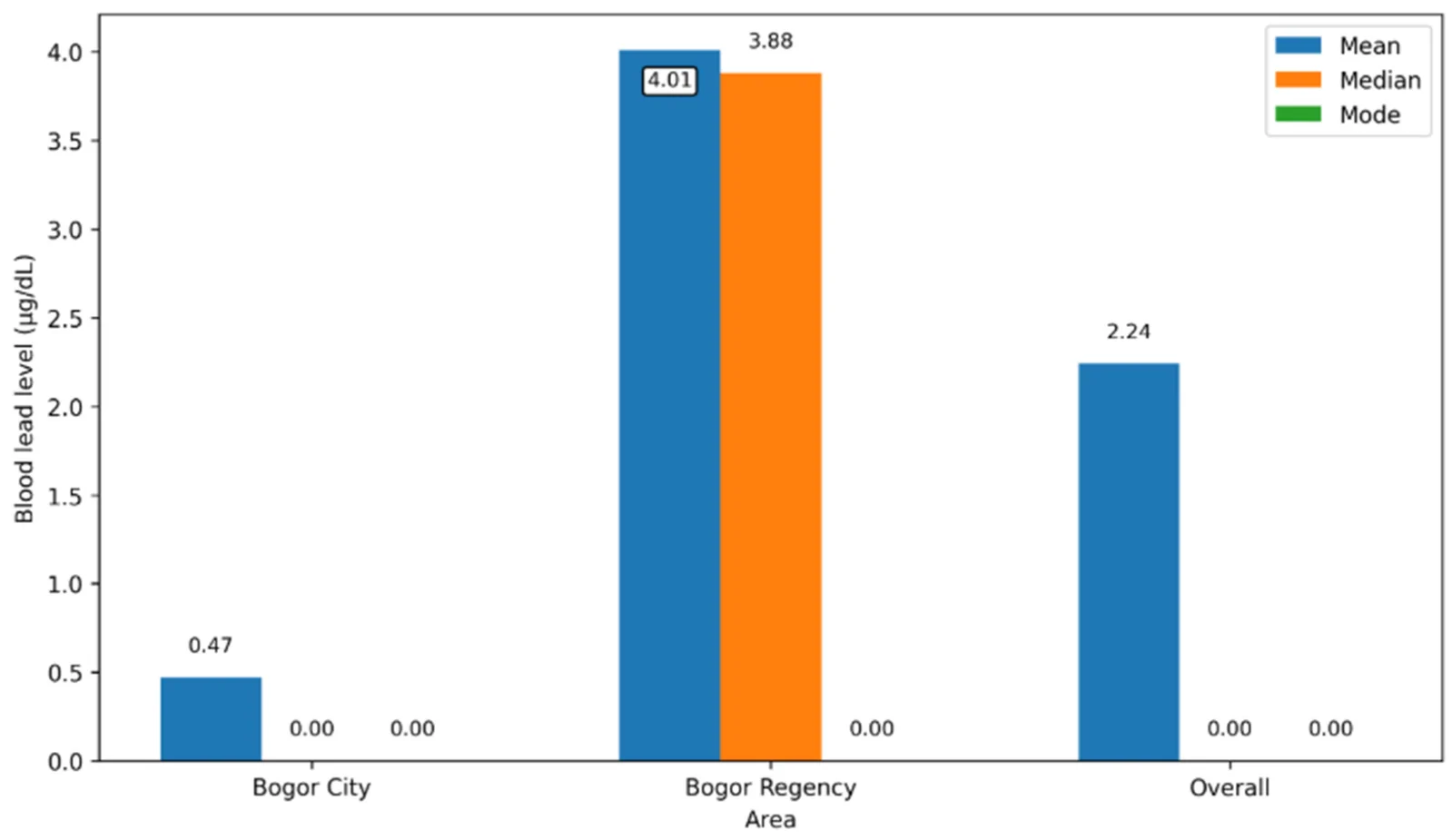
Comparison of mean, median, and mode of BLL by study area.

Figure 2 shows clear differences in blood lead level (BLL) distribution between Bogor City and Bogor Regency. Bogor Regency had a substantially higher mean BLL than Bogor City (4.01 µg/dL vs. 0.47 µg/dL), with a median BLL of 3.88 µg/dL, indicating that elevated values were more widely distributed among children in Bogor Regency. In contrast, Bogor City had a median and mode of 0.00 µg/dL, suggesting that most children had non-detectable BLLs, although one extreme value increased the mean.

Table 2 presents the distribution of BLL among children across primary health centers (PHCs) highlighting substantial geographic disparities. Facilities in Bogor Regency consistently show higher BLLs compared to those in Bogor City. The highest burden was observed in PHC Pasar Rebo, with a mean BLL of 6.93 µg/dL, median of 6.74 µg/dL, and 82.9% of children exceeding 5 µg/dL, indicating a severe exposure cluster. PHC Tenjo and PHC Pasir also demonstrated high mean BLL (5.65 and 6.42 µg/dL, respectively) and high proportions of elevated BLL (77.1% and 58.6%). Moderate levels were observed in PHC Rumpin and PHC Klapa Nunggal, while PHC Citeureup showed relatively low exposure (mean 0.39 µg/dL; 5.7% ≥ 5 µg/dL).

By contrast, facilities in Bogor City generally reported low BLL. Most centers had mean BLL below 1 µg/dL and 5.7% of children exceeded 5 µg/dL. An exception was PHC Pulo Armyn, which had a relatively higher mean BLL (2.03 µg/dL) and an extreme maximum value (66.82 µg/dL), suggesting a localized outlier case rather than widespread exposure.

Table 2 demonstrates a clear concentration of elevated BLL risk in Bogor Regency, particularly around Pasar Rebo, Tenjo, and Pasir, which correspond to known or suspected exposure sources of informal ULAB recycling. In contrast, no ULAB recycling activities were observed in Rumpin. However, extensive stone and sand mining operations were present throughout the area.

The observed geographic clustering suggests that local environmental sources may contribute to elevated BLL. However, because environmental observations were not analyzed as formal qualitative or exposure-measurement data, source attribution should be interpreted cautiously and requires confirmation through environmental sampling.

#### 3.1.4. Risk Factors for Elevated BLL

Bivariate analysis assessed associations between selected risk factors and elevated BLL (≥5 µg/dL). No significant associations were found for gender (*p* = 0.746), parental income (*p* = 0.744), peeling paint or household lead materials (*p* = 0.740), parental cosmetic use (*p* = 0.978), or aluminum cookware (*p* = 0.056).

In contrast, several factors were significantly associated with elevated BLL. Poor housing conditions, particularly limited access to clean water, were linked to higher BLL (*p* = 0.009). Children living with household members engaged in lead-related occupations also had higher prevalence (32.7% vs. 20.4%; *p* = 0.045).

The strongest associations were observed for environmental proximity and geographic location. Children living near lead-processing activities had substantially higher prevalence of elevated BLL (44.2% vs. 16.2%; *p* < 0.001). Similarly, prevalence was markedly higher in Bogor Regency compared with Bogor City (41.2% vs. 2.4%; *p* < 0.001) (Table 3).

Multivariable logistic regression analysis was conducted to identify independent predictors of elevated BLL. After adjustment for proximity to lead-processing activities, housing conditions, and the presence of household members engaged in lead-related occupations, geographic location emerged as the strongest independent determinant of elevated BLL. Children residing in Bogor Regency had substantially higher odds of elevated BLL compared with those in Bogor City (adjusted OR 25.124; 95% CI 9.713–64.989; *p* < 0.001), indicating marked spatial disparities in exposure risk. In addition, proximity to lead-processing activities remained a significant predictor, with children living near such sites having more than twice the odds of elevated BLL compared with those living farther away (adjusted OR 2.522; 95% CI 1.385–4.345; *p* = 0.002). In contrast, housing conditions and the presence of household members engaged in lead-related occupations were not statistically significant after adjustment, suggesting that their effects may be mediated through broader environmental exposure contexts (Table 4).

### 3.2. Follow-Up Actions After Elevated BLL Results

BLL results were returned to the participating facilities and caregivers. Ninety-two children had BLL ≥ 5 µg/dL and entered the guideline-based response pathway, which included counselling, exposure-reduction advice, nutritional support, and planned reassessment through the primary health center. The child with BLL 66.82 µg/dL received an urgent referral for specialist assessment and environmental/home investigation. Longer-term completion of repeat testing, referral, and treatment was not evaluated within this cross-sectional analysis.

### 3.3. Association Between BLL and Health Outcomes

Among 356 children assessed for anemia, prevalence was similar between those with BLL < 5 µg/dL (22.1%) and ≥5 µg/dL (22.5%), but there was no significant association (OR = 1.025; 95% CI: 0.550–1.911; *p* = 0.938). Among 411 children evaluated for wasting, prevalence was higher in the ≥5 µg/dL group (36.7%) compared to <5 µg/dL (30.6%), but this difference was not statistically significant (OR = 1.311; 95% CI: 0.803–2.141; *p* = 0.278) (Table 5).

Table 6 shows that anemia was observed in 27.1% of wasted children, compared with 14.8% of non-wasted children, and this association was statistically significant (*p* = 0.006). When stratified by BLL category, the association between wasting and anemia was not statistically significant among children with lower BLLs, although a similar trend was observed (24.4% vs. 15.0%; *p* = 0.062). In contrast, among children with elevated BLLs, anemia was substantially more frequent among wasted children than non-wasted children (35.2% vs. 14.3%), and the association was statistically significant (*p* = 0.030). These findings suggest that wasting is associated with anemia, with a potentially stronger relationship among children with elevated BLLs. However, given the cross-sectional design, causal interpretation should be avoided.

## 4. Discussion

This study provides a comprehensive assessment of childhood lead exposure within a primary health care–based implementation setting in Indonesia, using quantitative screening data supported by contextual environmental observations. The findings confirm that lead exposure remains a significant public health concern, with 21.9% of children presenting elevated BLL (≥5 µg/dL), despite a relatively low mean BLL. The observed right-skewed distribution, characterized by a small proportion of highly exposed individuals, is consistent with global evidence showing that localized environmental sources often contribute to high exposure clusters within otherwise lower-exposure populations [3,6].

### 4.1. Comparison with Global and Indonesian Evidence

The prevalence of elevated BLL observed in this study aligns with estimates from LMICs, where approximately one-third of children globally are estimated to have BLL ≥ 5 µg/dL [6]. In Indonesia, recent national and subnational data similarly indicate a substantial burden of exposure, particularly in areas with informal industrial activities such as ULAB recycling and small-scale mining [8,9,14]. Previous studies in Indonesia have reported elevated BLL among children living near ULAB recycling sites and industrial zones, with prevalence often exceeding 30–50% in high-risk areas [9,13,15].

The markedly higher prevalence observed in Bogor Regency compared with Bogor City is consistent with these findings and reflects the uneven distribution of environmental risk factors. This geographic disparity supports the growing body of evidence indicating that lead exposure in LMICs is highly context-specific, driven by proximity to pollution sources rather than broad socioeconomic indicators alone [1].

### 4.2. Environmental and Household Determinants

The study identified environmental proximity as a key determinant of elevated BLL. Children living near lead-processing activities had significantly higher odds of exposure, consistent with extensive literature demonstrating the impact of ULAB recycling, industrial emissions, and contaminated environments on childhood lead burden [5,16,17]. The multivariable analysis further highlighted geographic location as the strongest predictor, reinforcing the importance of spatial clustering and localized exposure sources.

An important observation from this study was the presence of mining-related activities in a high-prevalence area, suggesting a potential additional pathway for lead exposure. While informal ULAB recycling is widely recognized as a major source of lead contamination globally, the contribution of small-scale stone and sand mining has been less frequently reported [6]. Emerging evidence indicates that mining activities can mobilize naturally occurring heavy metals, generate contaminated dust, and increase environmental dispersion, potentially contributing to chronic exposure among nearby communities [18,19]. Although environmental observations in this study were used only as contextual information and not analyzed as formal exposure data, these findings highlight the need for broader environmental surveillance beyond traditional industrial sources in Indonesia.

Household-level factors also showed mixed associations. Limited access to clean water was significantly associated with elevated BLL, potentially reflecting broader environmental and infrastructural vulnerabilities that increase exposure risk. Similarly, children living with household members engaged in lead-related occupations showed higher exposure in bivariate analysis, consistent with the “take-home exposure” pathway described in occupational health literature [20]. However, these factors were not significant in multivariable models, suggesting that their effects may be mediated through environmental proximity and broader contextual exposures.

### 4.3. Non-Significant Findings and Interpretation

Several expected risk factors—including peeling paint, household lead-containing materials, parental cosmetic use, and aluminum cookware—were not significantly associated with elevated BLL in this study. These findings should be interpreted cautiously, particularly for peeling paint, as exposure assessment was based only on caregiver report without laboratory confirmation of lead content. Consequently, exposure misclassification may have occurred because not all peeling paint contains lead, and the presence of lead-based paint could not be objectively verified [20,21]. This may partly explain the absence of a significant association despite strong evidence from high-income settings identifying lead-based paint as an important exposure source. Similarly, the lack of association with cosmetics and cookware may reflect the low prevalence of unsafe product use or limitations of self-reported exposure measures. These findings underscore the importance of incorporating objective environmental and product testing in future studies to better characterize exposure pathways [22,23].

### 4.4. Health Outcomes

The study did not find significant associations between elevated BLL and anemia or wasting. While lead exposure has been linked to anemia through interference with heme synthesis and iron metabolism, evidence from epidemiological studies is mixed, particularly at lower exposure levels [12]. The relatively low mean BLL in this study may partly explain the absence of detectable associations. Additionally, anemia and wasting are multifactorial conditions influenced by nutritional status, infections, and socioeconomic factors, which may dilute the observable effect of lead exposure in cross-sectional analyses [12,24].

In addition to the separate analyses of BLL with anemia and wasting, the exploratory cross-tabulation suggested a clinically relevant overlap between wasting and anemia, particularly among children with elevated BLL. Among children with complete anemia and wasting data, anemia was more frequent among wasted than non-wasted children overall (27.1% vs. 14.8%; *p* = 0.006). When stratified by BLL category, this pattern was not statistically significant among children with BLL < 5 µg/dL (24.4% vs. 15.0%; *p* = 0.062), but was significant among children with BLL ≥ 5 µg/dL (35.2% vs. 14.3%; *p* = 0.030). These findings should be interpreted as exploratory because the study was not powered to test effect modification by BLL and because anemia and wasting share multiple underlying determinants, including inadequate nutrition, infection, inflammation, and socioeconomic vulnerability. Nevertheless, the pattern suggests that children with combined nutritional vulnerability and elevated BLL may represent a subgroup requiring closer follow-up within primary healthcare services. Geographical interpretation of anemia and wasting was not emphasized because facility-level stratification resulted in small cell counts and unstable estimates; larger studies with sufficient sample size and environmental measurement are needed to evaluate whether health outcomes cluster geographically with lead exposure.

### 4.5. Implications for Health System Integration

A key strength of this study lies in its implementation within routine primary health care services. The ability to conduct BLL screening through primary health centers suggests operational feasibility for embedding environmental health within existing health systems in LMICs. However, future studies should report more explicit implementation indicators—such as turnaround time, referral completion, fidelity, and acceptability—to better assess scalability and sustainability [25].

Furthermore, linking contextual environmental observations with clinical screening data provides a more comprehensive understanding of possible exposure pathways and may support more targeted, context-specific interventions. This approach is particularly relevant in settings such as Indonesia, where environmental monitoring systems are limited and fragmented.

### 4.6. Strengths and Limitations

This study benefits from implementation within routine health services and inclusion of diverse facility contexts. However, several limitations should be acknowledged. First, facilities were purposively selected and recruited similar numbers of children, limiting population generalizability. Second, household and environmental exposures were self-reported and no environmental lead measurements were performed. Third, building age and renovation history were not collected, and peeling paint was not tested for lead. Fourth, the association with limited clean water cannot be attributed to lead in water because water sources, plumbing, storage, and lead concentrations were not assessed. Fifth, area and proximity to lead-processing activities were likely correlated, but formal collinearity diagnostics and facility-clustered sensitivity analyses were not performed. Sixth, the cross-sectional design limits causal inference. Seventh, health outcomes were restricted to anemia and wasting and did not include neurodevelopmental, behavioral, neurologic, auditory, renal, or longitudinal growth outcomes. Finally, completion of repeat testing, referral, chelation, and environmental remediation occurred beyond the analytic period and was not evaluated.

### 4.7. Future Directions

Future research should incorporate longitudinal follow-up, individual-level BLL distribution analyses, standardized neurodevelopmental and behavioral measures, and systematic environmental sampling of paint, dust, soil, water, consumer products, and occupational take-home pathways. Building age and renovation history should be recorded, and drinking-water investigations should distinguish source-water contamination from premise plumbing and storage-related contributions. Larger studies should assess clustering by facility, evaluate collinearity between geographic variables, and measure completion and effectiveness of clinical and environmental interventions.

## 5. Conclusions

This study shows that childhood lead exposure remains an important public health concern in Indonesia, with 21.9% of children aged 1–5 years showing elevated BLL (≥5 µg/dL). Significant geographic disparities were identified, particularly in Bogor Regency and among children living near lead-processing activities, underscoring the importance of environmental proximity in shaping exposure risk.

The findings also suggest that blood lead screening can be integrated into routine primary health care services through the existing system, although stronger implementation measures are needed to assess its scalability and sustainability.

Overall, this study provides useful evidence to inform expansion of health system–integrated lead exposure management, including strengthened health screening, laboratory capacity, environmental follow-up, and cross-sectoral collaboration in Indonesia and other LMIC settings.

## Figures and Tables

**Table 1:** Participant characteristics.

Variables		Number	Percent
Gender	Female Male	198 222	47.1 52.9
Parents’ monthly income (IDR)	<3 million 3–10 million >10 million N/A	248 158 8 6	59.0 37.6 2.0 1.4
Resided near lead-processing activities, including ULAB recycling	No Yes	334 86	79.5 20.5
Housing conditions	Adequate Limited access to clean water Dirt floors Dirt floors and limited access to clean water	379 26 13 2	90.2 6.2 3.1 0.5
Peeling paint within the household (wall, furniture, etc.)	No Yes	231 189	55 45
Household members engaged in lead-related occupations	No Yes	368 52	87.6 12.4
Parents use cosmetics that are not labelled as safe	No Yes	409 11	97.4 2.6
Parents use aluminium cookware	No Yes	274 146	65.2 34.8
Pica Behaviour	No Yes	407 13	96.9 3.1

**Table 2:** Facility-level summary (ranked by % BLL ≥ 5 µg/dL).

City/Area	Facility (PHC)	Mean BLL	Median BLL	% BLL ≥ 5	Max BLL
Bogor Regency	Pasar Rebo	6.93	6.74	82.9	32.56
Bogor Regency	Tenjo	5.65	6.52	77.1	13.94
Bogor Regency	Pasir	6.42	6.6	58.6	18.66
Bogor Regency	Rumpin	2.72	0.0	38.9	13.51
Bogor Regency	Klapa Nunggal	1.84	0.0	31.4	9.57
Bogor Regency	Citereup	0.39	0.0	5.7	8.03
Bogor City	Kedung Badak	0.34	0.0	5.7	6.34
Bogor City	Lawang Gintung	0.18	0.0	2.9	6.47
Bogor City	Pulo Armyn	2.03	0.0	5.7	66.82
Bogor City	Warung Jambu	0.17	0.0	2.9	5.96
Bogor City	Bogor Selatan	0.10	0.0	2.9	3.8
Bogor City	Pondok Rumput	0.0	0.0	0.0	0.0

**Table 3:** Bivariate Analysis of Risk Factors for Elevated BLL (≥5 µg/dL).

Risk Factors		BLL (%)		*p* Value
		<5 µg/dL	≥5 µg/dL	
Gender	Female Male	154 (77.7) 174 (78.3)	44 (23.3) 48 (21.7)	0.746
Parents’ monthly income (IDR)	<3 million 3–10 million >10 million N/A	190 (76.6) 124 (78.5) 8 (100) 6 (100)	58 (23.4) 34 (21.5) 0 (0) 0 (0)	0.744
Housing Condition	Adequate Limited access to clean water Dirt floors Dirt floors and limited access to clean water	301 (79.4) 15 (57.7) 11 (84.6) 1 (50)	78 (20.6) 11 (42.3) 2 (15.4) 1 (50)	0.009
Household members engaged in lead-related occupations	No Yes	293 (79.6) 35 (67.3)	75 (20.4) 17 (32.7)	0.009
Peeling paint within the household (wall, furniture, etc.)	No Yes	179(77.5) 149 (78.8)	52 (22.5) 40 (21.2)	0.045
Resided near lead-processing activities, including ULAB recycling	No Yes	280 (83.8) 48 (55.8)	54 (16.2) 38 (44.2)	<0.001
Area	Bogor City Bogor Regency	204 (97.6) 124 (58.8)	5 (2.4) 87 (41.2)	<0.001
Parents use cosmetics that are not labelled as safe	No Yes	299 (97.4) 110 (97.3)	8 (2.6) 3 (2.7)	0.978
Parents use aluminium cookware	No Yes	192 (62.8) 82 (72.6)	115 (37.5) 31 (27.4)	0.056
Pica Behaviour	No Yes	111 (27.3) 2 (15.4)	296 (72.7) 11 (84.6)	0.341

**Table 4:** Adjusted Odds Ratios for Risk Factors of Elevated BLL.

Risk Factors		BLL		*p* Value
		Adjusted OR	95% CI	
Housing Condition	Adequate Limited access to clean water Dirt floors Dirt floors and limited access to clean water	1 0.920 1.278 10.429	0.420–2.762 0.140–5.345 0.240–453.268	0.798
Household members engaged in lead-related occupations	No Yes	1 0.945	0.440–2.029	0.612
Resided near lead-processing activities, including ULAB recycling	No Yes	1 2.522	1.385–4.345	0.002
Area	Bogor City Bogor Regency	1 25.124	9.713–64.989	<0.01

**Table 5:** Odds Ratios for Anemia (n = 356) and Wasting (n = 411) According to BLL Category.

	Anemia		OR	95% CI	*p* Value
**BLL** <5 µg/dL ≥5 µg/dL	No 222 (77.9) 55 (77.5)	Yes 63 (22.1) 16 (22.5)	1 1.025	0.550–1.911	0.938
	**Wasting**				
**BLL** <5 µg/dL ≥5 µg/dL	No 222 (69.4) 57 (63.3)	Yes 98 (30.6) 33 (36.7)	1 1.311	0.803–2.141	0.278

**Table 6:** Relationship between anemia, wasting status, and BLL category among children with complete data.

BLL Category	Wasting	Total Children n	Anemia		*p*-Value
			No n (%)	Yes n (%)	
<5 µg/dL	No	107	91 (85.0)	16 (15.0)	0.062
	Yes	160	121 (75.6)	39 (24.4)	
	Total	267	212 (79.4)	55 (20.6)	
≥5 µg/dL	No	35	30 (85.7)	5 (14.3)	0.030
	Yes	54	35 (64.8)	19 (35.2)	
	Total	89	65 (73.0)	24 (27.0)	
Overall	No	142	121 (85.2)	21 (14.8)	0.006
	Yes	214	156 (72.9)	58 (27.1)	
	Total	356	277 (77.8)	79 (22.2)	

## Data Availability

The data presented in this study are not publicly available due to ethical and institutional restrictions, including agreements with collaborating institutions and protection of participant confidentiality. Requests for access to de-identified data may be considered by the corresponding author in consultation with the Ministry of Health of Indonesia and UNICEF.

## Abbreviations

The following abbreviations are used in this manuscript:

ATSDR	Agency for Toxic Substances and Disease Registry
BLL	Blood Lead Level
ICP-MS	Inductively Coupled Plasma Mass Spectrometry
LMICs	Low- and Middle-Income Countries
PHC	Primary Health Center
QA/QC	Quality Assurance and Quality Control
ULAB	Used Lead–Acid Battery
